# Anodic oxide formation on aluminium-terbium alloys

**DOI:** 10.1007/s10008-016-3139-1

**Published:** 2016-03-16

**Authors:** Andrei Ionut Mardare, Carina Daniela Grill, Isabella Pötzelberger, Tanja Etzelstorfer, Julian Stangl, Achim Walter Hassel

**Affiliations:** Christian Doppler Laboratory for Combinatorial Oxide Chemistry (COMBOX) at the Institute for Chemical Technology of Inorganic Materials, Johannes Kepler University Linz, Altenberger Str. 69, 4040 Linz, Austria; Institute for Chemical Technology of Inorganic Materials, Johannes Kepler University Linz, Altenberger Str. 69, 4040 Linz, Austria; Institute of Semiconductor and Solid State Physics, Johannes Kepler University Linz, Altenberger Str. 69, 4040 Linz, Austria

**Keywords:** Combinatorial libraries, High-throughput experimentation, Scanning droplet cell, Anodic oxide film, Terbium

## Abstract

Aluminium terbium alloys were prepared by simultaneous thermal evaporation resulting in a thin film library covering a 5 to 25 at.% Tb compositional spread. Synchrotron x-ray diffraction (XRD) proves all of the alloys to be amorphous. Scanning electron microscopy (SEM) measurements reveal the structural changes upon increase in Tb content with the formation of small, Tb-rich segregations right before a drastic change in morphology around 25 at.% Tb. Anodic oxides were formed systematically in cyclic voltammograms using scanning droplet cell microscopy. Coulometric analysis revealed a linear thickness over formation potential behaviour with film formation factors ranging from 1.2 nm V^−1^ (5 at.% Tb) to 1.6 nm V^−1^ (25 % Tb). Electrochemical impedance spectroscopy was performed for each incremental oxidation step resulting in a linear relation between inverse capacity and formation potential with dielectric constants ranging from 8 (5 at.% Tb) to 16 (25 at.% Tb).

## Introduction

Aluminium is a very unnoble metal that easily forms oxides through the reaction with water or oxygen [1]. The oxide itself is transparent and colourless. In nature, however, the trigonal aluminium oxide that is called corundum, can appear coloured due to impurities; Fe^3+^ yields yellow and green colours, Fe^2+^ and Ti^4+^ causes blue colour, V^4+^ gives violet colour and Cr^3+^ caused colours ranging from pink to red, whereas the latter one is called ruby by definition. The reason for the unexpected colorization of, e.g. Cr^3+^ is the fact that it takes the place of the smaller Al^3+^ ion, which results in a strong ligand field splitting. A different mechanism of light absorption and emission is found for lanthanoids which have a partly filled 4f^n^ configuration that is efficiently shielded by the 5s^2^ and 5p^6^ shells, the latter one being influenced by the direct chemical environment. This electron configuration yields peculiarities in the absorption/emission behaviour, which are characterized by long life times of excited states due to the forbidden transitions and very sharp transitions due to the low interactions with the surrounding [2].

Terbium with its electron configuration of [Xe] 4f^9^ 6s^2^ is one of the so-called heavy rare earth elements that forms both trivalent and tetravalent cations, with the trivalent one being highly dominating in aqueous solutions. Some magnetic applications have been reported, e.g. for AlN-coated TbFe in magneto-optical media [3] or perpendicular magnetic recording of Tb in porous alumina [4] most of the applications, however, focus on its ability to specifically absorb and emit light. One way to produce Tb-doped oxide films was shown by Fang et al. They attached Tb chips to a ZnO target and obtained transparent conductive oxide films by RF sputtering that were doped to a level of 4.1 % [5]. Jia et al. used a wet chemical route with subsequent calcinations to produce Y- or Tb-doped ZnO. They found a strong influence on the structure that was changed from a nanorod-type for pure ZnO to a cluster or flake like appearance [6]. Meulenkamp and Kelly doped Ta_2_O_5_ films with Tb^3+^ and studied the dependence of the electroluminescence on the electrode potential in the presence of hydrogen peroxide [7]. Similar studies were conducted on alumina to detect and quantify Tb(III) chelates [8] or Luminol [9] at the electrode interface. These applications are only possible for thin valve metal oxide films in which the films form a dielectric barrier, are sufficiently chemically stable and having a decent band structure and band gap. The film must not be too thick, otherwise, the tunnelling probability will drop too much [10, 11] and not too thin, otherwise, the break down field strength would be exceeded [12]. Also, the number and energetic position of defects and dopants play a decisive role as they serve as centres for resonance tunnelling [13, 14].

Kulmala et al. performed a detailed study in which they ascribe the application to the hot electron injection through the insulating aluminium oxide film that acts as a tunnelling barrier for adsorbed species [15]. The observed luminescence was ascribed to a ^5^D_4_ → ^7^F_3_ transition of Tb(III) and a detailed energy scheme is given. Alternatively, porous alumina films can be used as shown by Staninski to electrogenerate chemiluminescence of adsorbed terbium compounds [16]. One step further goes the approach of Zalas and Klein using thin layers containing lanthanide ions to study dye-sensitized solar cells [17].

A more inherent link between terbium and aluminium is used in garnets such as Tb_3_Al_5_O_12_. These materials are of practical interest due to their high Verdet constant that can be used to switch the rotation axis of light in a magnetic field. Single crystals of that material were prepared by hybrid laser floating zone machining [18].

Such doped films can be also prepared by a combustion synthesis in which aluminium nitrate, together with urea and the nitrate of either terbium or thulium forms lanthanide-doped alumina films that are prone to thermoluminescence [19]. Further, it is also possible to obtain a doped nanoporous alumina film through a corresponding anodisation procedure in an electrolyte of proper composition [20].

Spray pyrolysis is another method to prepare terbium-doped alumina films as it was shown by Esparza-Garciá et al. [21] along with their luminescence characterisation. Martínez-Martínez et al. on the other hand used the same method to study the white light emission of Ce^3+^, Tb^3+^ and Mn^2+^ codoped alumina films exhibiting a high conversion efficiency of up to 85 % [22] for specific potentials.

Theoretical attempts towards the energetics of trivalent cations in γ-Al_2_O_3_ were made by Maglia and Gennari which calculated interatomic potentials through energy minimization using an Al_64_O_96_-defect spinel supercell containing eight aluminium vacancies [23].

All of these studies rely on the use of specific recipes; in this sense, they are all lacking a systematic concentration variation. The present work aims at a comprehensive study and systematic understanding of the influence of composition in mixed aluminium terbium oxides formed anodically. In this inaugural work, the preparation, along with a physical and electrochemical characterisation of the parent metal library and the anodically formed oxides, will be described.

### Experimental details

The Al-Tb thin film combinatorial library was fabricated on borosilicate glass substrates with sizes of 26 × 76 mm^2^ (VWR International GmbH) using a state-of-the-art, self-developed thermal co-evaporation unit with a base pressure of 5 × ×10^−4^ Pa. Before their use in vacuum, the substrates were cleaned using sequential ultrasonication in acetone, isopropanol and water. Two thermal sources, positioned off-centre in respect to the substrate, were simultaneously used for evaporation of Al and Tb, respectively. High purity Al and Tb metals (99.95 %, Goodfellow) were each placed in contact with thermal elements externally powered by high direct current sources (3.3 kW) individually controlled via LabView software. Al was evaporated directly from a W boat at an average power of 150 W while Tb was placed inside a BN crucible heated by a W coil requiring in excess of 300 W. The source-substrate distance for both sources was 120 mm and the control of individual evaporation rates was achieved in situ using quartz crystal monitors (QCMs, Inficon). Each QCM directly faces each evaporation source and the amount of crossed-over material being detected by one crystal was minimized by proper line-of-sight shielding. Self-developed LabView PID software was used for adjusting the power on each source according to the desired evaporation rate. Al was evaporated at a rate of 1.2 nm s^−1^, while Tb evaporation rate was defined at only 0.3 nm s^−1^. With these values, the calculated composition at the middle of the substrate was Al-10 at.% Tb in order to obtain a compositional gradient with low Tb content. The evaporation rates calculation was based on Al and Tb cosine laws defining their individual thickness distribution along the substrate. During the evaporation, the amount of heat delivered by radiation to the substrate was minimal, the end temperature reaching 373 K while the final chamber pressure was in the low 10^−3^ Pa. A final thickness of the Al-Tb library of 570 nm was calculated by summing the individual Al and Tb thickness values measured by their corresponding QCMs (470 and 100 nm, respectively).

The electrochemical study on the Al-Tb thin film combinatorial library was performed using scanning droplet cell microscopy (SDCM) [24]. A small electrolyte droplet partially released at the tip of a glass capillary comes in direct contact with the investigated sample (working electrode, WE). A full three-electrode microelectrochemical cell is obtained by using capillary-based μ-reference electrodes (μ-RE) and Au wire/bands counter electrodes (CE) enclosed in the SDCM body [25]. The current work used a μ-AuHg/Hg_2_(CH_3_COO)_2_/NaCH_3_COO RE with a potential of 0.4 V vs SHE, and its fabrication details can be found elsewhere [26]. The SDCM was operated in contact mode, when the electrolyte is perfectly confined within the capillary walls by using a soft silicone sealing at the cell’s tip and pressing it down with a controlled force [27]. In this way, a highly reproducible wetted area is ensured [24]. The entire Al-Tb thin film combinatorial library was automatically scanned along the compositional gradient (*x* axis) using a gantry robot, providing a compositional resolution of 2 at.%. The addressed area was measured by colour anodisation of a Ti surface followed by its observation using optical microscopy combined with image recognition software. Each addressed spot on the Al-Tb compositional spread surface had an area of 0.33 mm^2^.

All electrochemical experiments were done in borate electrolyte buffered at pH 9.0 using a CompactStat potentiostat (Ivium Technology) electrochemical system. The electrolyte was obtained by mixing aqueous solutions of H_3_BO_3_ (0.1 mol L^−1^) and NaOH (0.03652 mol L^−1^). Anodisation of individual alloys along the Al-Tb library was performed potentiodynamically with a rate of potential increase of 100 mV s^−1^. The maximum potential applied during each cyclic voltammogram (CV) was stepwise increased in 1 V steps up to 10 V vs the RE. Before each step of anodic oxide thickness increase, electrochemical impedance spectroscopy (EIS) was performed at 0 V in a maximum frequency range of 10^5^ to 10^−1^ Hz using a superimposed AC perturbation of 50 mV.

Energy-dispersive x-ray (EDX) was used for determining the compositional gradient along the Al-Tb thin film combinatorial library. The surface microstructure was observed by scanning electron microscopy (SEM; Zeiss Gemini 1540 XB) as a function of composition. The surface topography of the Al-Tb thin film library was investigated by atomic force microscopy (AFM) using a NanoSurf easyScan 2 system.

The crystallographic properties of individual Al-Tb alloys were mapped along the compositional gradient using synchrotron based diffraction experiments. Typical Ω-2θ powder scans were obtained at beamline BM20 at the European Synchrotron Radiation Facility in Grenoble with an energy of 10.3 keV. The scans were performed in the 2θ range of 1 to 80 °. The penetration depth was calculated for each addressed Al-Tb alloy and its value was in the order of the film thickness. However, for better visibility of the scattering signal stemming only from the Al-Tb thin film combinatorial library, an Ω-2θ scan obtained on the empty borosilicate glass substrate was subtracted from each measured diffractogram. This was performed taking into account the absorption of the overlaying thin film by subtracting between 95 and 78.5 % of the plain glass scan from At-Tb alloys containing 5 to 25 at.% Tb, respectively.

## Results and discussion

**Fig. 1:**
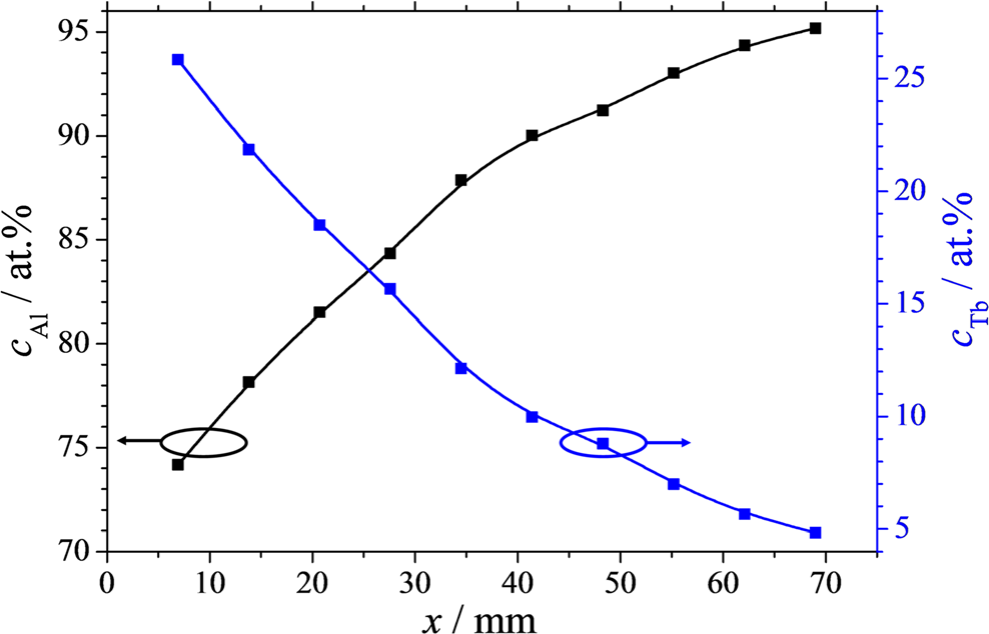
Composition vs position plot of the material library investigated in this study. Scanning electron microscopy within build scanning energy dispersive x-ray measurement was used to determine the local composition in each spot. A mechanical scan option of automatic sample movement was used with respect to the sample dimension

Before anodisation of individual alloys along the Al-Tb thin film combinatorial library, a compositional mapping was done using EDX. The primary electron beam was focused on various locations on the *x* axis of the sample along the compositional gradient while the positions (in mm) were recorded. The obtained compositional spread is presented in Fig. 1. A total compositional spread of approximately 20 at.% was obtained, with a central composition very close to the desired one of Al-10 at.% Tb. The calculated composition is based on a simple mixed matter model using the Al and Tb molar volumes and assuming that all atoms released from each source will condense on the substrate, their local surface density depending on each relative source-substrate position on both horizontal and vertical planes. The good correlation between the calculated compositional value and the experimentally obtained one indicates that both Al and Tb have comparable sticking coefficients and volatility while the Al atoms scattering by the much larger Tb is minimized. This is not always the case when mixing two species in vapour phase and strong scatterings may be observed [28]. However, in the present case, the much smaller number of Tb atoms (as compared to the Al) combined with low thermal energy per atom allowed an almost ideal atomic mixture. The non-linear character of the EDX composition mapping is directly related to the cosine thickness distribution above each source resulting in the present case in a composition variation ranging between Al-5 at.% Tb and Al-26 at.% Tb.

**Fig. 2:**
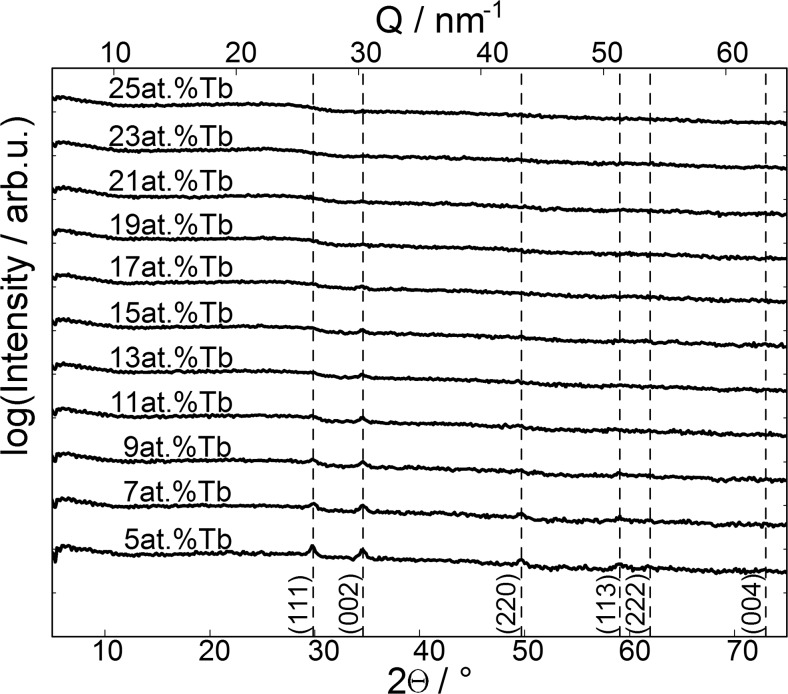
2*Θ* x-ray diffraction scans obtained at different positions along the Al-Tb gradient. The *bottom x*-axis denotes 2*Θ*/° at the used energy of 10.3 keV. The *top x*-axis shows the momentum transfer *Q* in reciprocal space which reads *Q*_z_ = 4 π/*λ* sin*Θ* for the used geometry. Slight contributions of various Al Bragg peaks could be observed. The evolution of the appearance corresponds well with the findings in SEM investigations, as discussed in the text

For characterizing individual crystallographic properties of Al-Tb thin film alloys, synchrotron radiation was used and Ω-2θ scans were performed at 11 different positions along the Al-Tb gradient ranging from Al-5 at.% Tb to Al-25 at.% Tb. In the typical Ω-2θ scans, the angle between the incident beam and the sample (Ω) varies up to half the value of the angle between the incident and the exit beam (2θ) while scanning. In symmetric coplanar diffraction geometry, this results in varying the incidence and diffracted beam angles equally, similar to Bragg-Brentano geometry. Figure 2 shows the performed diffraction scans at different Al-Tb concentrations. At low Tb concentrations, the FCC structure typical to Al is evidenced by the presence of representative diffraction peaks indicated in the figure. Increasing the Tb concentrations resulted in a gradual loss of the overall crystallinity as observed from the decrease of the peak intensities with the amount of Tb. Up to 9 at.% Tb, several FCC peaks can still be observed suggesting a cubic structure for the corresponding alloys. Above 9 at.% Tb only a weak (002) peak may be observed which gradually fades as the Tb concentration approaches 19 at.%. Above 19 at.% Tb, no diffraction peaks could be identified anymore. This amorphisation may be related to the big difference between Al and Tb atomic sizes. Since the library deposition is performed by thermal evaporation at room temperature, the single atom energy is low, in the range of 0.1 eV. This actually may trigger an almost instant ‘freezing’ of the surface species upon condensation. However, the smaller Al atom still retains a low amount of energy which is used in surface diffusion triggering the formation of a cubic structure when no or low Tb amounts are present. At high Tb concentrations, the heavier Tb atom acts as surface scattering centres impeding the formation of a crystalline structure by disrupting the natural surface diffusion of Al and its FCC phase nucleation.

**Fig. 3:**
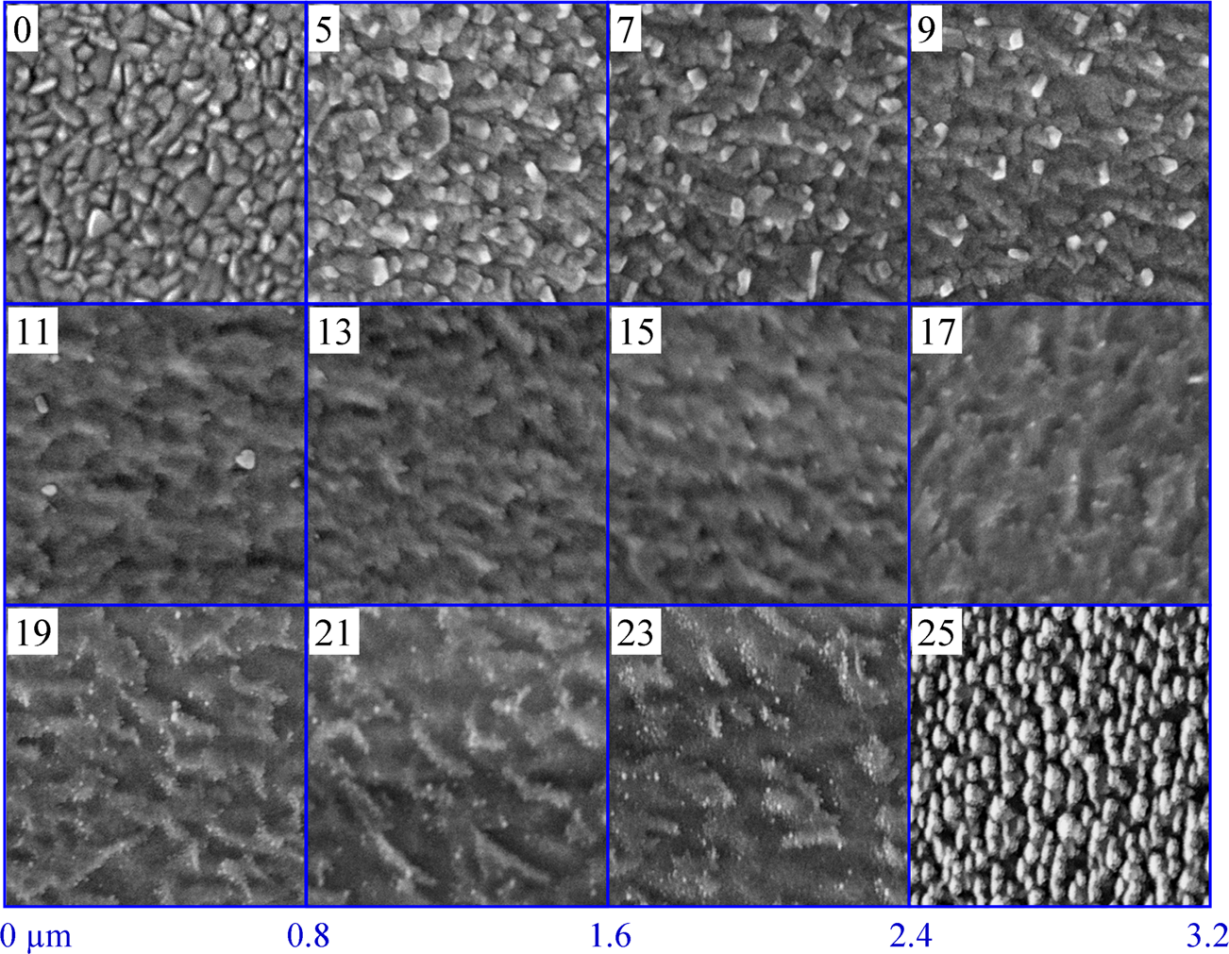
Scanning electron microscopic images at different positions of the material library. The local concentration of terbium in this region is given in at.% as an *inset* in the *upper left corner* of each subimage. For comparison, an SEM image of a pure aluminium film prepared under identical conditions is shown in the *left upper part* of the composed figure

The surface microstructure of the Al-Tb thin film combinatorial library was investigated by scanning electron microscopy (SEM) before and after performing the electrochemical study. A tableau containing SEM images of the Al-Tb surface before anodisation is presented in Fig. 3. As inlets, the amount of Tb (in at.%) is indicated in each image, the Al amount being complementary. As a reference, the surface of a pure Al thin film deposited under identical conditions as the Al-Tb library is shown indexed as 0 at.% Tb in the upper left corner of Fig. 3. The SEM images of the Al-Tb surface after oxidation (not shown here) did not present any significant differences when compared with those from Fig. 3. This is most likely due to the secondary electrons tunnelling through the very thin anodic oxide during the image acquisition resulting in imaging of the underlying parent metal surface. The anodisation process can ultimately lead to a certain degree of smoothening of the original metallic surface, which is not the case in this study due to the rather low anodisation potentials used. Scanning the library resulted in imaging the microstructure as a function of the Tb concentration with a compositional resolution of 2 at.%. The presence of 5 at.% Tb in the library results in a slight deviation of the appearance from that of the pure Al surface. The Al grains start to elongate reaching approximately 100 nm and partly stick out from the surface due to the Tb presence. This is better observed for higher Tb amounts up to 9 at.% where a distinct SEM contrast can be observed at smaller grains of approximately 50 nm uniformly distributed along the investigated surface. The evolution of these small grains coincides with a distortion of the underlying larger grains (initially resembling the pure Al) which form a quite smooth support for the 50-nm grains with undefined grain boundaries. At 11 at.% Tb, the entire surface is completely transformed and the smaller grains disappear. The surface only shows the transformed underlayer. This coincides with the compositional threshold triggering the disappearance of the cubic (111) and (220) peaks, as evidenced by previous XRD experiments, suggesting that the small 50-nm grains may be responsible for the diffraction peaks. Further increasing the Tb amount in the Al-Tb compositional spread, the surface microstructure features are conserved until Al-19 at.% Tb where a new surface emerges. The underlayer starts to be decorated with extremely small grains in the range of 10 nm and this composition coincides with the complete amorphisation of the Al-Tb library discussed before. Additions of up to 23 at.% Tb in the library do not produce a considerable surface change, only the number of decorated grains is slightly increasing. The last image presented in Fig. 3 corresponding to the highest Tb amount of 25 at.% shows a completely new surface totally changed as compared to slightly lower Tb amounts. This dramatic change may be due to reaching the solid solution compositional threshold of 25 at.% Tb [29] beyond which Al_3_Tb may form. However, still no crystallinity could be observed for this composition during the XRD investigations. One may speculate that this slight exceeding of the phase boundary already causes a visual conversion but still with extremely small grains that do not contribute to a sufficient diffraction signal.

**Fig. 4:**
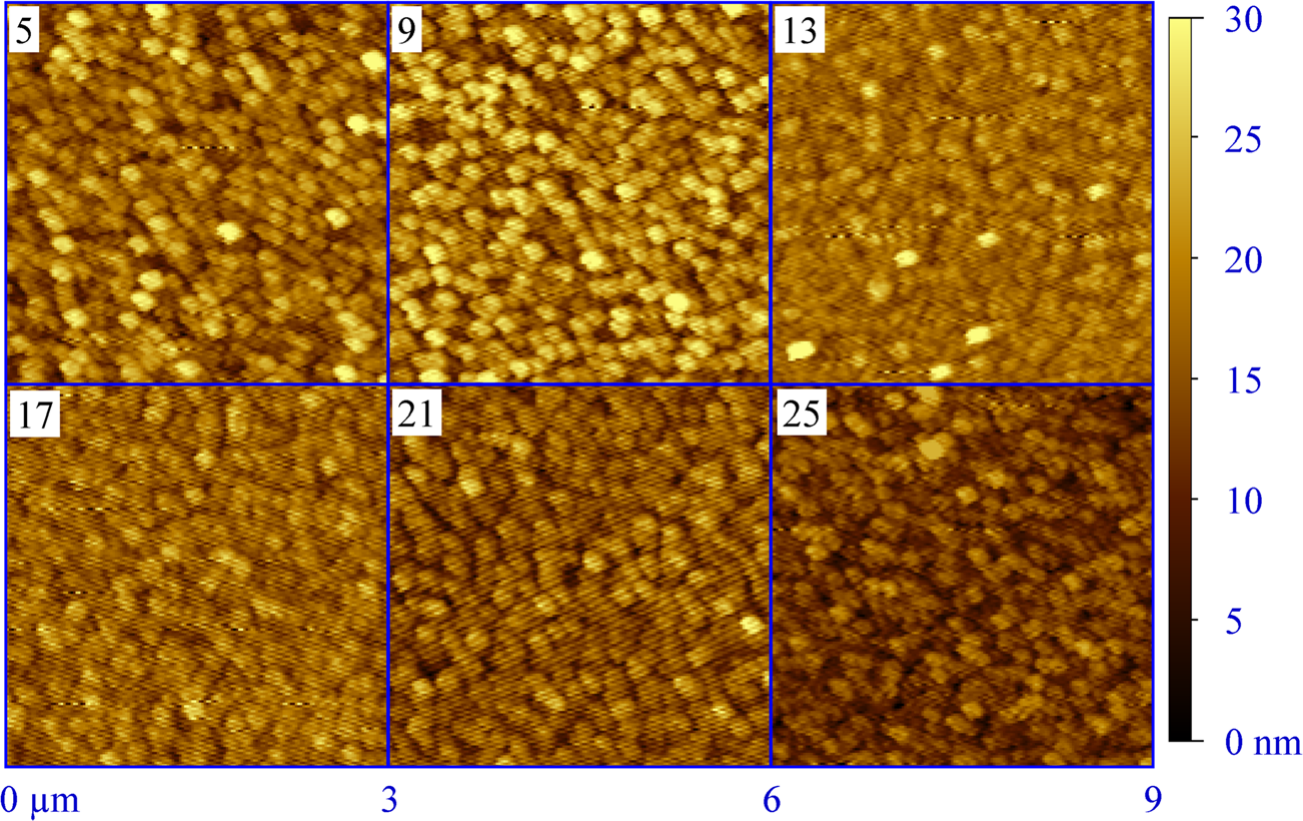
AFM topographic surface imaging of various Al-Tb alloys along the thin film library. The Tb concentration in at.% is given for each image

Before performing any electrochemical study, a correct measurement of the wetted area at every addressed spot/alloy must be performed. Such study is not trivial, since a correct evaluation would be based not only on surface topography but also on electrolyte surface wetting behaviour at nanometre scale as a function of the library composition. However, assuming that the entire exposed surface of the parent metal is wetted by the electrolyte upon SDCM contact, a calculation of the real surface area would be sufficient. In order to achieve this goal, AFM imaging of the Al-Tb compositional spread surface was performed. In Fig. 4, the topographies of several selected Al-Tb compositions are presented using the same height scale/range of 0–30 nm. Each figure was obtained by scanning a 3 × 3 μm^2^ area and the amount of Tb in at.% is given for each image. The topography evolution along the Al-Tb compositional spread does not show significant changes. Surface grain clusters with equivalent diameters of approximately 150 nm can be observed in every case. The previously discussed smoothening at the nanometre scale of the library surface in the middle of the analysed compositional spread (see Fig. 3) is hinted here for 13 and 17 at.% Tb by a decrease of the height range. However, the real electrolyte addressed area can only be estimated by using a common algorithm for surface measurements. In the present work, the real area was calculated using the triangulation method [30] and the obtained results are summarized in Table 1. The results indicate that the real surface deviates from the projected one with maximum 2 % for the entire Al-Tb library, which falls into the experimental error of the AFM investigations. Therefore, the projected area of 0.33 mm^2^ was used for all further electrochemical investigations.

**Table 1:** Ratios between the calculated real surface area and the projected (geometrical) area measured by AFM for selected Al-Tb thin film alloys

*c*_Tb_/at.%	5	9	13	17	21	25
*A*_Real_/*A*_Geom_	1.0164	1.0231	1.0117	1.0208	1.0173	1.0133

**Fig. 5:**
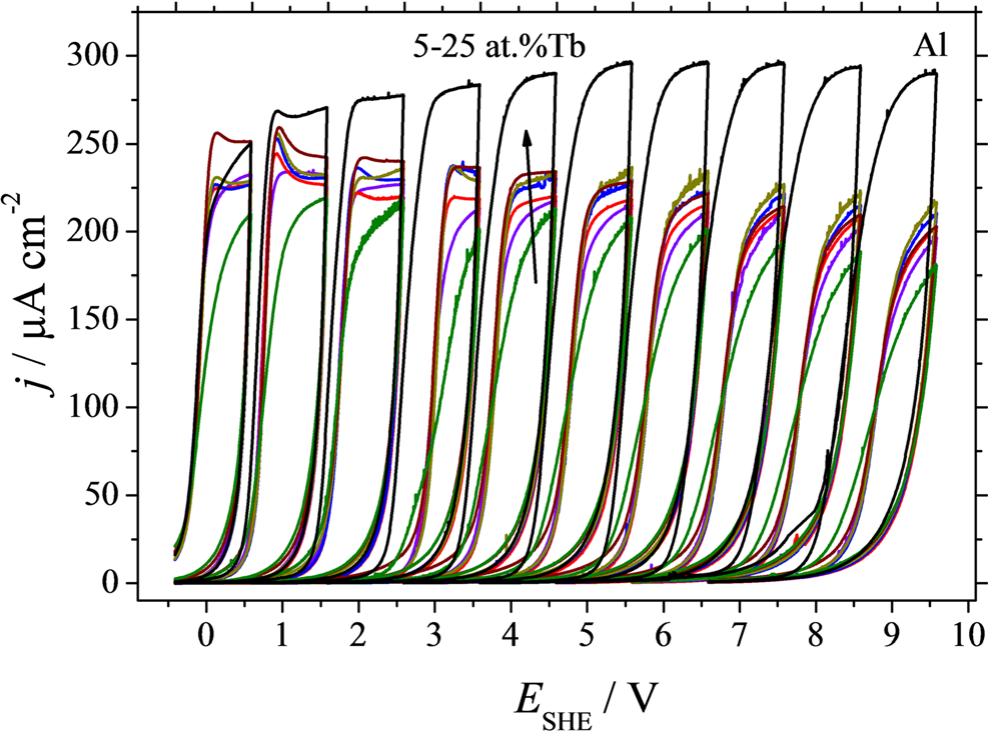
Selected sets of potentiodynamic cyclovoltamograms. Each single spot corresponding to a defined composition was scanned electrochemically up to a maximum potential and scanned back to the initial potential. The maximum potential was increased stepwise to yield a complete series. The series with the highest current density given in *black* refers to the pure aluminium film. The sets with Tb concentrations of 5, 10, 15, 20, and 25 at.% are given in *different colours*. The *arrow* in the *central set* indicates the increasing Tb content

The electrochemical anodisations in the Al-Tb thin film combinatorial library were performed potentiodynamically with high compositional resolution along the entire compositional gradient. Series of cyclic voltammograms were obtained at each Al-Tb alloy addressed by the SDCM. In Fig. 5, a few examples of such series are presented (for avoiding an overcrowded appearance of a full presentation). Additionally, the series obtained during anodisation of a pure Al film under the same conditions are also plotted as a reference. In all cases, a typical valve metal behaviour can be observed, characterized by a strong current rectification upon electric field weakening on the reverse potential scan. The oxide is grown in steps of 1 V and during each anodisation step, a current density plateau may be seen. The valve metal behaviour of Al-Tb alloys is clearly triggered by Al, as observable from the continuous change of the CVs shape with increasing Tb amount. This situation is similar to Al-Fe [31] or Al-Cu [32] alloys. Anodisation of pure Al results in a steady current density plateau during each oxide growth step. The presence of 5 at.% Tb changes this behaviour dramatically. The current density drops by approximately 30 % but still, a valve metal behaviour is present. Increasing the Tb amount in the Al-Tb thin film library resulted in a slow increase of the current density plateaus as indicated by the arrow added in Fig. 5 to describe Tb concentrations between 5 and 25 at.% (with an equidistant step of 4 at.%). The current density plateaus can be directly attributed to the oxide film formation factors (given in nm V^−1^) using Faraday’s law and the high field model of anodic oxide growth on valve metals [33]. Figure 5 suggests a sudden drop of the oxide formation factors from pure Al to Al-5 at.% Tb. Slowly increasing the Tb concentration results in an increase of the oxide formation factors and a flattening of the current density plateaus, but the pure Al levels are not reached.

**Fig. 6:**
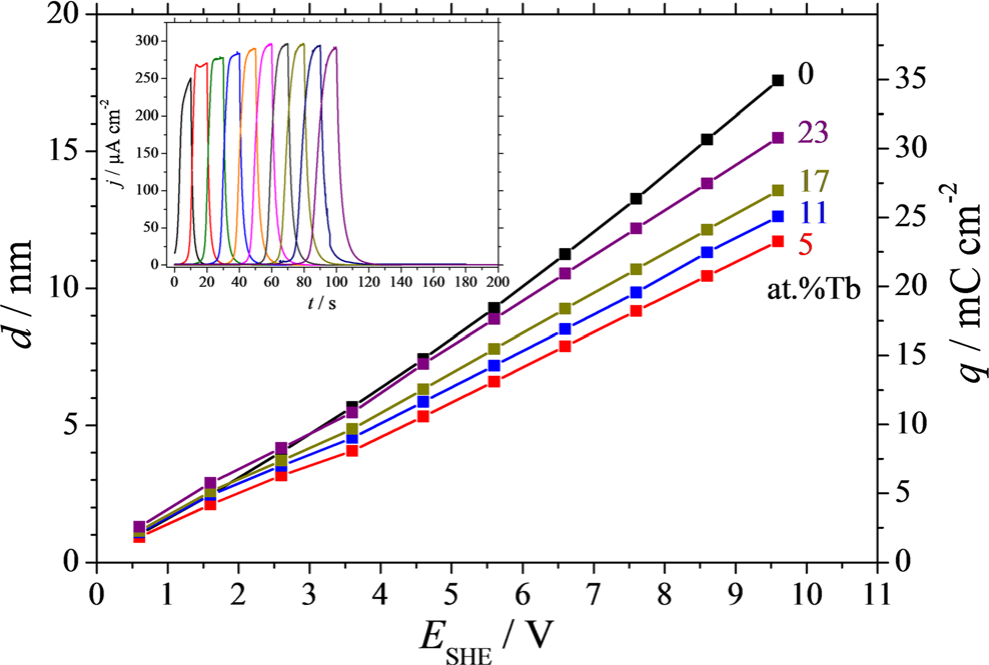
Film thickness and consumed electrochemical charge (inlet) of anodic oxides measured after each 1 V step in the anodisation potential for various compositions of the Al-Tb thin film alloys

In order to quantify the individual oxide formation factors along the Al-Tb compositional spread, the charge consumed during each oxide formation step must be calculated. Integration of the CVs plotted as a function of time allows a direct charge density calculation. Several representative plots of the cumulative charge density as a function of the applied potential are presented in Fig. 6. The measured charge density is directly related to the actual thickness of the oxide (Faraday law), if oxide specific parameters are known (e.g. density, molar mass, number of exchanged electrons), which is presented as the left side vertical axis in Fig. 6. For this calculation, individual mixed oxide densities and molar masses were evaluated as functions of components concentration by means of a linear distribution between the densities or molar masses of pure Al_2_O_3_ and Tb_2_O_3_, according to the mixed matter theory. In Table 2, a few calculated oxide density and molar mass values used for the calculation of the thicknesses plotted in Fig. 6 are summarized. An example of the CVs plotted vs time is given in the inlet of Fig. 6 for pure Al for illustration. Integration of each curve (corresponding to each incremental oxidation scan step up to 10 V) results in a charge density amount whose cumulative values are plotted in Fig. 6. For all presented cases, a quasi-linear dependence of the charge density on applied potential can be observed. The higher slope corresponding to the pure Al as compared to the Al-Tb alloys indicates again a higher oxide formation factor for pure Al. Addition of 5 at.% Tb to Al leads to a strong decrease of the corresponding curve slope which starts to increase again with the Tb amount.

**Table 2:** Mixed oxide density and molar masses calculated along the Al-Tb library using the mixed matter theory by linear extrapolation between the values of pure Al_2_O_3_ (*c*_Tb_ = 0) and pure Tb_2_O_3_ (*c*_Tb_ = 100)

*c*_Tb_/at.%	0	5	11	17	23	100
*ρ*_ox_/g cm^−3^	3.5	3.72	3.98	4.25	4.51	7.91
*M*_ox_/g mol^−1^	101.96	115.15	130.98	146.82	162.65	365.85

**Fig. 7:**
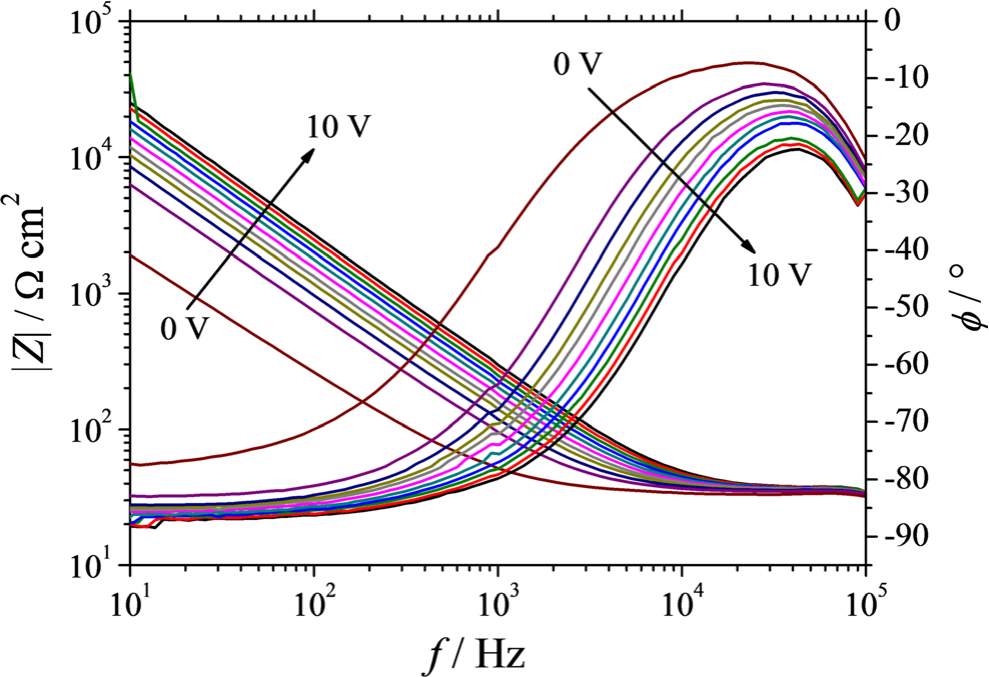
Typical Bode plots of the impedance measurements performed on Al-Tb thin film combinatorial library after anodic oxide growth at various maximum applied potentials. The *curves* exemplified were measured for Al-25 at.% Nb

**Fig. 8:**
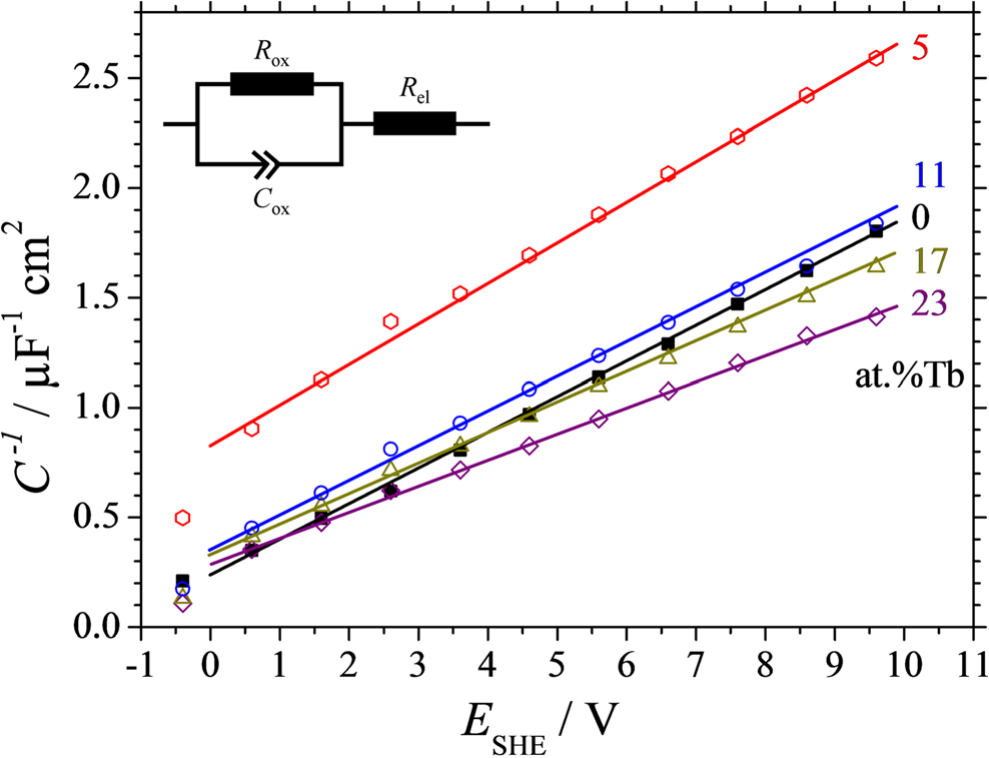
Inverse capacitance of anodic oxides measured after each 1 V step in the anodisation potential for various compositions of the Al-Tb thin film alloys

After each step of oxide growth, EIS was performed in order to analyse the electrical behaviour of individual alloys along the Al-Tb thin film combinatorial library. In Fig. 7, a typical series of Bode plots obtained from the frequency-dependent impedance and phase shift measurements is presented as measured for Al-25 at.% Nb. The final anodisation potential was increased from 0 to 10 V vs RE as suggested by the arrows in the graph. In all cases, a pure capacitive behaviour was observed, with the phase shifting toward −90 ° below 10^3^ Hz. An expected increase of the impedance as a function of oxide thickness is observable and the −1 slope of the impedance spectra suggest a typical RC-R equivalent circuit. Fitting all the EIS curves using such equivalent circuit allowed a direct measurement of the anodic oxide capacitances. In Fig. 8, the values of the inverse capacitances obtained at selected compositions are plotted as a function of the applied potential. The equivalent circuit used for fitting the EIS data is presented as an inlet. The slopes of the inverse capacitance curves will directly describe the oxide electrical permittivities since the potential axis is related to the oxide thickness through the oxide formation factor. Similarly to the charge density plots, the plot corresponding to the anodic oxide grown in identical conditions on pure Al is presented as reference. The highest slope observable belongs to Al-5 at.% Tb and is constantly decreasing with the increase of the Tb content along the Al-Tb compositional spread. The lowest slope, indicating the highest dielectric constant, is observed for 23 at.% Tb in Fig. 8.

**Fig. 9:**
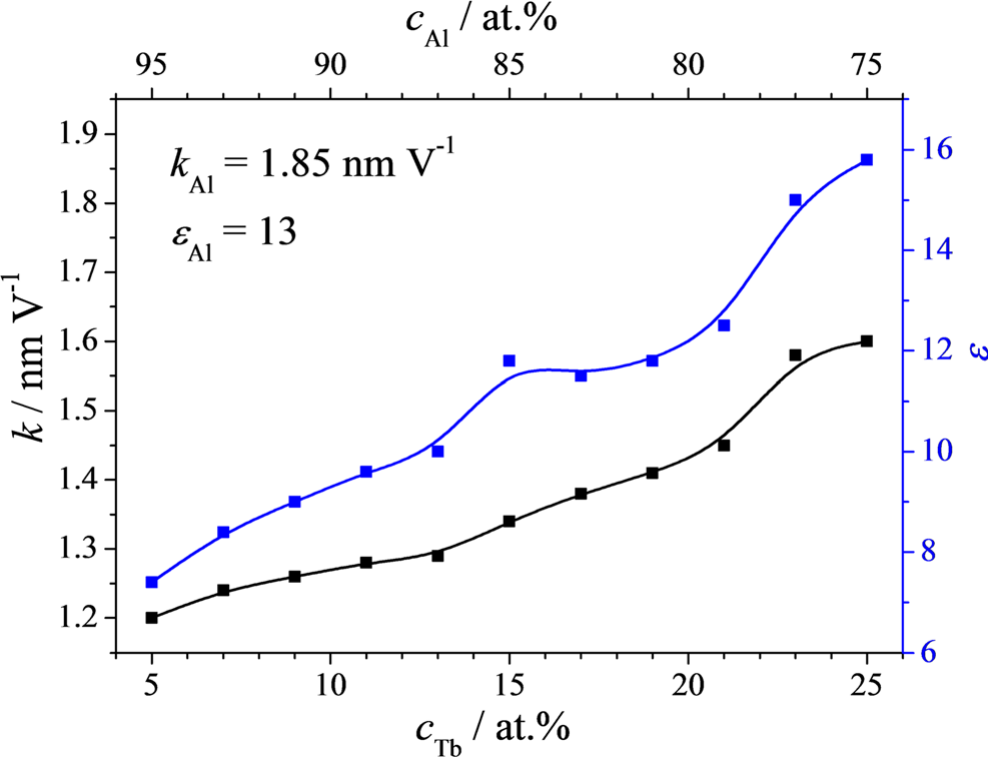
**a** Oxide formation factors and dielectric constants for the anodic oxides grown on the Al-Tb combinatorial library

In Fig. 9, the final results obtained from Figs. 6 and 8 are summarized. A complete oxide formation factor and dielectric constant mapping of the analysed Al-Tb thin film combinatorial library is presented. Both Al and Tb concentrations are provided as complementary horizontal axis on the top and bottom, respectively. Additionally, the values measured on anodic oxides grown in the same conditions on pure Al are provided in the figure. Starting from a formation factor of 1.85 nm V^−1^ on pure Al, a strong drop is observed at 5 at.% Tb, as previously hinted from Fig. 5, where the anodic oxide forms approximately 1.2 nm per each applied 1 V. Increasing the Tb amount led to a slow increase of the oxide formation factor toward a maximum value of 1.6 nm V^−1^ measured for 25 at.% Tb. The evolution of the dielectric constant along the Al-Tb compositional spread follows the same trend. From a value of 13 measured on anodized pure Al, the lowest Tb concentration in the library resulted in a value below 8. Increasing the Tb amount in the library leads to an increase of the dielectric constant until the value of pure Al_2_O_3_ is surpassed and a value of almost 16 is measured for Al-25 at.% Tb.

All electrochemical results obtained in this study must be interpreted from the point of view of the used electrolyte. According to the Al-Tb phase diagram, in the presented compositional spread, Al-Tb forms solid solutions. In such case, for high alloying concentrations, it is expected that Al tends to impose its anodisation behaviour on the alloy. However, this cannot be generalized as a rule. A particularly difficult aspect of the present work was finding a suitable electrolyte pH where both Al and Tb are stable. Unfortunately, since the Pourbaix diagram of Tb indicates its highest stability at a pH value around 9.0, the Al stability will be decreased [1]. This fact is directly observable in the curves corresponding to pure Al showing much higher current density plateaus in Fig. 5 and a higher oxide formation factor (Fig. 8) of 1.85 nm V^−1^ as compared with value typically obtained in pH 6.0 of 1.6 nm V^−1^ [10]. The apparent increase of the *k* factor for pure Al is attributed to an expected partial dissolution of Al in pH 9.0 decreasing the anodisation current efficiency. Anodisation of the entire Al-Tb library was also performed using a pH 6.0 acetate-buffered electrolyte where Al exhibits high stability. In that case, the Al-Tb alloys completely dissolved during the anodisation process rendering the entire electrochemical data unusable. In spite of the much higher Al concentration, the Al-Tb library instability is most likely due to the Tb instability at this pH. Therefore, a compromise was found in using a pH value where Tb is stable, i.e. 9.0. This additional experiment with pH 6.0, even though unsuccessful, indicated that the Tb reaction to the pH changes is much accentuated as compared to the Al behaviour. Therefore, as far as the Al-Tb library is concerned, the Tb presence is expected to increase the current efficiency by stabilizing the alloy in pH 9.0. Future work is planned on these materials to study the amount of dissolved Al and/or Tb via flow-type SDCM coupled with downstream analytics (e.g. inductively coupled plasma mass spectrometry) [34, 35] as well as the electronic structure of the oxides and their possible electroluminescence and photoluminescence behaviour [36].

## Conclusions

A systematic investigation of Al-Tb alloys requires an approach different from a one at a time study. Simultaneous thermal evaporation of the parent metals Al and Tb from decentred crucibles allows preparing lateral composition graded libraries ranging from 5 to 25 at.% Tb. Even small amounts of terbium (5 at.%) are sufficient to yield a completely amorphous metallic film. The anodic oxidation behaviour is strongly influenced by the Tb content. Film formation factor and dielectric constant of the dielectric films measured using a pH 9.0 electrolyte are significantly suppressed by small amounts of 5 at.% Tb with a tendency to recover upon increase to 25 at.%. Terbium has a strong amorphisation effect on the parent metallic alloy as was proven by synchrotron XRD.
